# Translation validation of a new back pain screening questionnaire (the STarT Back Screening Tool) in French

**DOI:** 10.1186/0778-7367-70-12

**Published:** 2012-06-07

**Authors:** Olivier Bruyère, Maryline Demoulin, Clara Brereton, Fabienne Humblet, Daniel Flynn, Jonathan C Hill, Didier Maquet, Julien Van Beveren, Jean-Yves Reginster, Jean-Michel Crielaard, Christophe Demoulin

**Affiliations:** 1 Department of Public Health, Epidemiology and Health Economics, University of Liege, Liege, Belgium; 2 Department of Modern and Foreign Languages, Faculty of Philosophy and Letters, University of Liege, Liege, Belgium; 3 English language trainer for adults, Verviers, Belgium and Médecins sans frontières, Brussels, Belgium; 4 Arthritis Research UK Primary Care Centre, Keele University, Stoke-on-Trent, UK; 5 Department of Motricity Sciences, University of Liege, Liege, Belgium; 6 Department of French and Romance Languages and Literatures, Faculty of Philosophy and Letters, University of Liege, Liege, Belgium

**Keywords:** Low back pain, Questionnaire, Translation

## Abstract

**Background:**

Low back pain (LBP) is a major public health problem and the identification of individuals at risk of persistent LBP poses substantial challenges to clinical management. The STarT Back questionnaire is a validated nine-item patient self-report questionnaire that classifies patients with LBP at low, medium or high-risk of poor prognosis for persistent non-specific LBP. The objective of this study was to translate and cross-culturally adapt the English version of the STarT Back questionnaire into French.

**Methods:**

The translation was performed using best practice translation guidelines. The following phases were performed: contact with the STarT Back questionnaire developers, initial translations (English into French), synthesis, back translations, expert committee review, test of the pre-final version on 44 individuals with LBP, final version.

**Results:**

The linguistic translation required minor semantic alterations. The participants interviewed indicated that all items of the questionnaire were globally clear and comprehensible. However, 6 subjects (14%) wondered if two questions were related to back pain or general health. After discussion within the expert committee and with the developer of the STarT Back tool, it was decided to modify the questionnaire and to add a reference to back pain in these two questions.

**Conclusions:**

The French version of the STarT Back questionnaire has been shown to be comprehensible and adapted to the French speaking general population. Investigations are now required to test the psychometric properties (reliability, internal and external validity, responsiveness) of this translated version of the questionnaire.

## Background

Low back pain (LBP) is a major public health problem as it is the most prevalent and costly musculoskeletal problem in today’s economically advanced societies, and may lead to long-term disability combined with frequent use of health services [1]. The natural course for most patients with non-specific LBP is that symptoms are self-limiting within a few weeks, but some patients develop persisting LBP [2]. Although a rapid decrease in pain and incapacity often occurs within the first month following the onset of pain [3,4], estimates suggest that 23% of patients experience persistent symptoms of whom 11-12% report substantial levels of disability [5]. It is these more disabled individuals who account for the vast majority of the socioeconomic impact of LBP [6]. Primary care evidence-based guidelines for non-specific back pain highlight the importance of identifying indicators of poor prognosis in order for treatment to be appropriately targeted [7,8]. Indeed, there is growing evidence that a better identification of prognostic indicators leads to more effective early prevention treatments for back pain in primary care [2,9,10].

A few questionnaires have been developed to predict long term disability and failure to return to work [5]. The Orebro Musculoskeletal Pain questionnaire, developed by Linton et al. in 1997, is one of the most well-known. More recently, the STarT Back Screening Tool (SBST) was developed and validated to identify subgroups of patients to guide the initial decision making in primary care [11]. This tool is based on the presence of potentially modifiable physical and psychological indicators for persistent, disabling symptoms, identified through nine questions. Patients are classified as “low risk” of future disabling LBP if they score positively on fewer than four questions. The remainder are then subdivided into “medium risk” (physical and psychosocial indicators of poor outcome, but without high levels of psychological indicators) and “high risk” (high levels of psychological prognostic indicators with or without physical indicators). Interestingly, this tool has good psychometric capacity and is shorter than the Orebro Musculoskeletal Pain questionnaire [12]. Recently, a large randomised controlled trial involving 851 adults followed for 12 months compared the clinical effectiveness and cost-effectiveness of stratified primary care (using the SBST questionnaire) with non-stratified current best practice [13]. The results demonstrated that the stratified care approach significantly reduced levels of disability and was cost-saving compared to the current best practice management approach.

The SBST was developed in England and the translation has only been cross-culturally validated in Spanish [14] and Danish [15]. Currently, a French version of the tool has not yet been validated. The cross-cultural adaptation of a health status self-administered questionnaire for use in a new country, culture and/or language requires a unique methodology in order to reach equivalence between the original source and the target language [16,17]. The objective of this study was to translate and culturally adapt the SBST into French.

## Methods

The cross-cultural adaptation went through seven phases according to guidelines [16].

### Phase 1: Contact with SBST developers

A contact was made with the team, in England, that originally validated the English version of the questionnaire. The objective was to inform them about the project and to ask for their collaboration and approval.

### Phase 2: Initial translations (English to French)

Two forward translations were made from English into French independently of each other. Both translators were bilingual with French as their first language, and one having a medical background. The translators provided a written report with comments to highlight challenging phrases or uncertainties and the rationale for specific linguistic choices made.

### Phase 3: Synthesis

A synthesis of the original questionnaire and both initial French translations was performed, resulting in Version 1. The method involved comparing and noting translation discrepancies which reflected potentially ambiguous wordings. Inappropriate wording choices were identified and resolved following discussion between the translators and a written report made of this synthesis process, with actions taken to address and resolve issues that arose.

### Phase 4: Backward translations

Two translators (blind to the original version of the SBST) then independently translated Version 1 back into English. These translators had English as their first language and had no medical background. The objective of these backward translations was to make sure that Version 1 reflected the same item content as the original version.

### Phase 5: Expert committee review

An expert committee compared the backward translations with each other and with the original questionnaire. Differences in translation, and whether these reflected linguistic imprecisions or cultural differences, were debated with alternative wording suggested when needed. This expert committee included two methodologists, one health professional, one French language professional, and the four translators (forward and back translators) involved in the process. Telephone and email contacts with the developer of the SBST questionnaire were also made. This phase resulted in a pre-final version of the French translation of the SBST questionnaire and in a full written report of the issues at each step.

### Phase 6: Test of the pre-final version

The French version of the questionnaire was tested on 44 subjects with LBP randomly selected from a Spine Unit at the Liège University Hospital Centre, a private physiotherapy clinic and a fitness centre. Each subject completed the SBST and was questioned about any difficulties encountered in completing the questionnaire or understanding the purpose or meaning of each question. Following the interview process, the expert committee discussed the findings and proposed the final version.

### Phase 7: Final version

The final version of the SBST questionnaire was submitted, along with all available reports and forms to the developer of the instrument.

## Results

During phase 2, minor linguistic differences between the two forward translations emerged from all but two questions. They were observed for “spread down” (item 1), “I have had pain” (item 2), “it’s not really safe” and “to be physically active” (item 5), “worrying thoughts” (item 6), “I feel” and “it’s never going to get any better” (item 7), “in general I have not enjoyed all the things I used to enjoy” (item 8), “overall” and “bothersome” (item 9). Some differences were also observed for the introduction sentence (i.e. “thinking about” and “tick your response to the following questions”). These differences were discussed during phase 3 where all of these issues were resolved.

After the backward translation stage, the expert committee met to finalise the pre-final questionnaire. All items of the questionnaire were discussed; a few minor discrepancies occurred and were related to linguistic difficulties with “back pain”, “my leg(s)”, “enjoy”, “how bothersome” and “worrying thoughts”. Consensus on the translation of item 6 was the most difficult because “worrying thoughts have been going through my mind a lot of the time” has no real equivalence in French. The new version adopted by the expert committee could be translated in English as “I have often been concerned”. Consensus was therefore reached by the experts committee on all items to get the pre-final version of the questionnaire. The expert committee believed that no further cross-cultural adaptation was needed.

**Figure 1:**
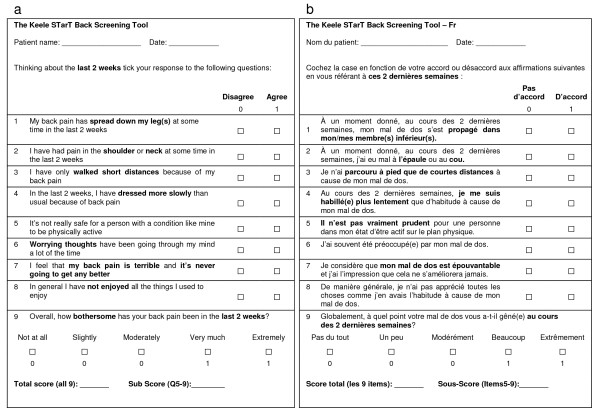
**a The English original version of the STarT Back questionnaire. b The French translated version of the STarT Back questionnaire.**

This pre-final version was subsequently tested in phase 6 on 44 patients with LBP, 59% were female, with a mean age of 48 (13.1) years (range 18 to 78 years). The participants interviewed indicated that all items of the questionnaire were clear and easy to understand. However, 6 subjects (14%) wondered if items 6 and 8 were related to back pain alone or their general health. After discussion within the expert committee and with the developer of the SBST, it was decided to modify the questionnaire to ensure these questions were clearly related to the back pain problem. Figure 1 provides the original English version and the French translated final version of the SBST.

## Discussion

With the increase in the number of international research projects, the need to adapt health status measures for use in other than the source language is of primary importance. The SBST questionnaire has been developed recently but is increasingly used in the country of origin [12,13,18-21]. A recent high profile article reported the relevance and benefits of a stratified care approach to low back pain management based on the SBST with matched pathways [13]. The objective of this study was to translate this questionnaire from English into French by following the international guidelines recommendations [16,17]. All items had a 100% response rate and no subjects experienced difficulty completing the questionnaire.

The strengths of this study include the use of three centres in various locations to recruit a broad spread of patients with LBP with a wide age distribution and good representation of both sexes. The main limitation of this study was that it was only performed in the French speaking part of Belgium. While we believe that this version of the questionnaire could be used without further adaptation in most of the French speaking countries in the world (e.g. Belgium, France, Switzerland, etc.) it remains possible that a cultural adaptation of the questionnaire could still be needed in other French speaking countries outside of Europe.

Translation difficulties encountered as part of this study included the fact that some English words and sentences from the original SBST were hard to translate into French (e.g. the sentence “worrying thoughts have been going through my mind a lot of the time”). However, a translation was identified that was considered to be equivalent. There was also considerable discussion regarding the translation of the word “leg”. The scientific translation is “membre inférieur” but current common parlance is to use the term “jambe” which refers, from a “scientific point of view” only to the lower part of the leg. It was decided to keep with the more scientific term “membre inférieur”, and interestingly, no patient expressed any difficulties with this choice of wording.

It should be acknowledged that while this formal translation process has provided useful insights into how a person interprets each questionnaire item, it did not address the construct validity, reliability, or item response patterns necessary for a successful cross-cultural adaptation [16,17]. Consequently, additional testing of the psychometric properties of the French version of the SBST questionnaire is necessary and is currently being planned. There are many ways in which translated questionnaires could be tested for their psychometric comparability with the source version. The objective is to ensure that the new version has demonstrated the measurement properties needed for the intended application. For example, strong evidence of construct validity is needed (i.e. is it measuring what it is supposed to be measuring?). In addition to construct validity, test-retest reliability (do the scores stay the same when the patients have not changed?) and responsiveness (ability to detect a change when it has occurred) is also of primary importance when assessing change over time. Finally, a further important step in the validation of this French version of the SBST will be to confirm the efficiency of this questionnaire alongside a matched treatment approach to test the effectiveness of stratified LBP management within a French speaking patient population.

## Conclusions

In conclusion, the translation of the SBST questionnaire was shown to be linguistically accurate and acceptable for use by French speaking patients in Belgium. This French version of the SBST is easy to understand and quick to complete and, when fully validated, will be of potential interest for the French speaking medical and scientific community.

## Competing interests

J. Hill was involved in the development of the initial English questionnaire.

## Authors’ contributions

OB, MD and CD were involved in the design of the study. OB drafted the manuscript. All authors participated to the translation process. All authors read approved the final manuscript.
